# Current algorithm for the surgical treatment of facial pain

**DOI:** 10.1186/1746-160X-3-30

**Published:** 2007-07-25

**Authors:** Konstantin V Slavin, Hrachya Nersesyan, Mustafa E Colpan, Naureen Munawar

**Affiliations:** 1 Section of Stereotactic and Functional Neurosurgery, Department of Neurosurgery, University of Illinois Medical Center at Chicago, Chicago, Illinois, USA

## Abstract

**Background:**

Facial pain may be divided into several distinct categories, each requiring a specific treatment approach. In some cases, however, such categorization is difficult and treatment is ineffective. We reviewed our extensive clinical experience and designed an algorithmic approach to the treatment of medically intractable facial pain that can be treated through surgical intervention.

**Methods:**

Our treatment algorithm is based on taking into account underlying pathological processes, the anatomical distribution of pain, pain characteristics, the patient's age and medical condition, associated medical problems, the history of previous surgical interventions, and, in some cases, the results of psychological evaluation. The treatment modalities involved in this algorithm include diagnostic blocks, peripheral denervation procedures, craniotomy for microvascular decompression of cranial nerves, percutaneous rhizotomies using radiofrequency ablation, glycerol injection, balloon compression, peripheral nerve stimulation procedures, stereotactic radiosurgery, percutaneous trigeminal tractotomy, and motor cortex stimulation. We recommend that some patients not receive surgery at all, but rather be referred for other medical or psychological treatment.

**Results:**

Our algorithmic approach was used in more than 100 consecutive patients with medically intractable facial pain. Clinical evaluations and diagnostic workups were followed in each case by the systematic choice of the appropriate intervention. The algorithm has proved easy to follow, and the recommendations include the identification of the optimal surgery for each patient with other options reserved for failures or recurrences. Our overall success rate in eliminating facial pain presently reaches 96%, which is higher than that observed in most clinical series reported to date

**Conclusion:**

This treatment algorithm for the intractable facial pain appears to be effective for patients with a wide variety of painful conditions and may be recommended for use in other institutions.

## Background

The term "facial pain" encompasses variety of clinical conditions ranging from very common (such as headaches and myofascial pain syndromes) to less common (trigeminal neuralgia – TN) and quite rare (glossopharyngeal neuralgia – GPN) (Table 1). Although clinical presentation of some of these conditions overlaps, the treatment approaches differ significantly based on etiology, nature and severity of pain, as well as its distribution, neurological and psychological variables, and medical co-morbidities [1]. The diagnostic uncertainty was discussed in multiple publications previously and remains problematic [2-4] (Table 2).

**Table 1:** Common categories of facial pain

Headache
Tension headache
Migraine and equivalents
Cluster headache
Non-neurogenic pain
Sinusitis
TMJ pain
Trigeminal neuralgia
Idiopathic TN (TN type 1)
Secondary TN (symptomatic TN)
Atypical trigeminal neuralgia (TN type 2)
Trigeminal neuropathic pain
Trigeminal injury (unintentional, incidental trauma)
Postherpetic neuralgia
Anesthesia dolorosa/trigeminal deafferentation pain (intentional deafferentation)
Other cranial neuralgias and other clinical syndromes
Glossopharyngeal neuralgia
Sudden unilateral neuralgiform pain with conjunctival injection and tearing
Eagle syndrome (elongated styloid process)
Tolosa-Hunt syndrome (cavernous sinus/orbital apex inflammation)
Gradenigo syndrome (petrous apex syndrome)
Ramsay-Hunt syndrome (herpetic infection of nervus intermedius)
Raeder (paratrigeminal) syndrome
Cancer-related facial pain
Atypical facial pain (somatoform pain disorder)
The list presented here is given for purposes of illustration of the algorithm described in this article. It is by no means comprehensive and the order of conditions does not reflect their incidence or severity.

**Table 2:** Burchiel's classification scheme for facial pains commonly encountered in neurosurgical practice (modified from Burchiel, 2003 [3])

**Pain category**	**History/Pain pattern**	**Other names**
Trigeminal neuralgia type 1	Spontaneous onset (>50% episodic pain)	Idiopathic trigeminal neuralgia
Trigeminal neuralgia type 2	Spontaneous onset (≥50% constant pain)	Atypical trigeminal neuralgia
Trigeminal neuropathic pain	Trigeminal injury - unintentional (trauma, sinus surgery)	
Trigeminal deafferentation pain	Trigeminal injury – intentional deafferentation (after destructive procedures)	Anesthesia dolorosa/hypesthesia dolorosa
Symptomatic trigeminal neuralgia/MS	Multiple sclerosis	
Symptomatic trigeminal neuralgia/other causes	Posterior fossa mass lesions, Chiari malformation	Secondary trigeminal neuralgia
Postherpetic neuralgia	Trigeminal *Herpes Zoster *outbreak	
Atypical facial pain	Somatoform pain disorder	

As to the available procedures for treatment of the medically intractable facial pain, the spectrum of interventions includes a wide variety of procedures starting from non-destructive approaches (such as microvascular decompression (MVD) of trigeminal and other cranial nerves) to percutaneous interventions (radiofrequency (RF) gangliolysis, glycerol injection, balloon compression) to less invasive destructive procedures (stereotactic radiosurgery) and to neuromodulation surgeries (peripheral and central neurostimulation) as well as central destructive surgeries (tractotomy, etc.) (Table 3.)

**Table 3:** Summary of surgical procedures for treatment of facial pain

Procedure name	Surgical details	Current status	Notes
Microvascular decompression	Retromastoid craniectomy, decompression of trigeminal nerve root from offending vessel(s)	Widely accepted (Jannetta procedure)	Non-destructive nature Requires general anesthesia Immediate improvement of pain
Radiofrequency gangliolysis	Percutaneous needle procedure; thermal destruction of trigeminal ganglion and root	One of the most established options for TN	Destructive procedure Intended to be very selective May be done with sedation Requires patient cooperation Immediate improvement of pain
Glycerol gangliolysis	Percutaneous needle insertion; chemical destruction of trigeminal fibers	Very commonly used percutaneous procedure	Destructive procedure May be selective No need in general anesthesia Immediate improvement of pain
Balloon compression	Percutaneous needle insertion; mechanical destruction of trigeminal fibers	Commonly used percutaneous procedure	Destructive procedure Non-selective May be done under general anesthesia Does nor require patient cooperation Immediate improvement of pain
Stereotactic radiosurgery	Focused radiation aimed at the trigeminal nerve root	Accepted treatment option	Destructive procedure Non-selective No need in general anesthesia Improvement of pain may take several months
Neurectomy	Surgical removal or interruption of peripheral branch of the trigeminal nerve	Rarely used option	Destructive procedure Highly selective Does not require general anesthesia Results in complete numbness of the area Immediate improvement of pain
Peripheral nerve stimulation	Electrical stimulation of the peripheral branch of trigeminal nerve	Relatively new application	Non-destructive procedure Involves trial before implantation Adjustable/reversible
Motor cortex stimulation	Electrical stimulation of motor cortex with electrode inserted into epidural space through small craniotomy	Considered "off label" indication in the US	Non-destructive procedure Requires craniotomy and general anesthesia Adjustable/reversible
Trigeminal tractotomy	Percutaneous or open surgical destruction of the nucleus caudalis in the upper spinal cord	Very rarely used treatment option	Destructive procedure High risk of complications Immediate improvement of pain

Recently, two in-depth clinical articles presented different modalities used in treatment of facial pain [5,6]. One of them reviewed a single-institution experience with two most commonly done procedures that are used for surgical treatment of the trigeminal neuralgia [5]. The other provided comprehensive review of currently available neurostimulation techniques that may be and are used for the treatment of facial pain [6]. In this paper, we reviewed our single-institution experience with the treatment of the facial pain according to our definition of multimodality approach.

Since our clinical practice attracts a large number of patients with facial pain, we decided to create an algorithm for its surgical treatment and summarize applicability of such algorithm based on degree of pain relief and patient satisfaction. The analysis presented here was derived from a prospective study of patients with facial pain that was approved by our Institutional Review Board few years ago [7].

## Methods

The algorithm for treatment of medically intractable facial pain that we use is based on taking into account underlying pathological processes, the anatomical distribution of pain, pain characteristics, the patient's age and medical condition, associated medical problems, the history of previous surgical interventions, and, in some cases, the results of psychological evaluation. The largest part of our algorithmic approach to facial pain is, not surprisingly, dedicated to treatment of trigeminal pathology, including TN and other disorders of trigeminal system (trigeminal neuropathy, deafferentation, etc.). Surgeries for migraine and other headache syndromes are currently in an early research phase; dental and TMJ problems rarely come to neurosurgical attention but should be kept as a part of differential diagnosis in patients with chronic facial pain; non-trigeminal disorders (related to involvement of the glossopharyngeal, intermediate and occipital nerves) are significantly less common (at least in our practice) and, in most cases, may be diagnosed by specific distribution of pain that involves ear, throat, occiput, neck alone or in addition to facial territory.

Importance of detection of the underlying pathological processes may be illustrated by difference in surgical management of various possible causes of symptomatic (secondary) TN (neoplasms, vascular malformations, etc.) that would require definitive treatment (such as tumor resection) in order to obtain pain control [8], or symptomatic TN caused by demyelination (as in multiple sclerosis (MS)) for which destructive procedures are considered to be a preferred treatment approach. In most cases of typical TN (TN types 1 and 2), where underlying pathology cannot be clearly identified, neurovascular compression is postulated to be a cause of pain (so called idiopathic TN), non-destructive procedure – microvascular decompression (MVD) – may be considered a treatment of choice, as this modality (a) provides the only curative solution, (b) is not associated with sensory loss as a result of surgery, (c) has very high success rate, and (d) is associated with the lowest rate of pain recurrence.

Patient's age and medical condition become important in algorithmic approach to facial pain, particularly in those diagnosed with TN, since MVD requires craniotomy and carries certain risk of complications related to surgery and general anesthesia. This risk increases with the patient's age and associated co-morbidities. Therefore, for older and medically unfit patients, preference is given to less invasive percutaneous destructive procedures and stereotactic radiosurgery (SRS).

Although MVD has been shown effective in elderly TN patients and those with secondary TN from MS, we do not routinely suggest it for these patient categories. This approach may reflect our institutional bias, but in the authors' opinion, the lower yield of MVD in MS and higher risk of complications in elderly patients undergoing MVD do not justify considering MVD as the first choice in these particular situations.

Among percutaneous destructive procedures that are used for TN, each of three modalities (RF gangliolysis, glycerol injection, and balloon microcompression) may be used for idiopathic (TN types 1 and 2) and symptomatic (secondary) TN, but in our opinion, RF gangliolysis works best in those TN patients that present with pain in 2^nd ^or 3^rd ^division of the trigeminal nerve (maxillary and mandibular nerves) due to somewhat higher risk of corneal hypesthesia or anesthesia with resultant keratitis and corneal abrasions if 1^st ^branch (ophthalmic nerve) fibers are targeted by RF energy.

Therefore, balloon microcompression in our algorithm is reserved for patients with TN whose pain involves distribution of the first trigeminal branch (ophthalmic nerve) with or without other branches.

SRS with Gamma Knife (or linear accelerator) is reserved for those patient with TN types 1 and 2 (typical and atypical TN) who prefer to avoid any mechanically invasive procedures (open or percutaneous interventions) and only if they can tolerate their pain for 1–3 months that are required for pain relief after radiosurgery.

The nature of pain and results of neurological evaluation allow one to diagnose other pain syndromes involving the face and differentiate them from classic TN (TN type 1). For example, presence of constant pain, lack of response to carbamazepine, and associated numbness would put the patient either into category of "atypical TN (ATN)" (if sharp and shooting pain is present) or "trigeminal neuropathic pain (TNP)" (where pain is predominantly constant and the neurological deficit is more obvious). Both of these conditions may be classified as TN type 2 (if the pain is idiopathic), true TNP (if caused by unintentional or incidental trauma) or trigeminal deafferentation pain (if caused by previous deafferenting procedures – surgeries, injections, etc.) [3,4]. MVD for patients in this category may be less effective than for those with TN type 1 (typical TN) and destructive procedures will have limited value only (this is true about both percutaneous procedures and SRS). For TN type 2 (ATN), the vascular compression is thought to be more distal, but for TNP in its broad definition (that includes neuropathic, deafferentation and post-stroke pains) the pain most likely represents partial or complete deafferentation from previous injury (either iatrogenic or traumatic) or cerebral infarction (thalamic strokes, lateral medullary infarctions – Wallenberg syndrome). The limited choices of intervention include (a) peripheral nerve stimulation (PNS) [9,10] that works particularly good in TNP patients, (b) trigeminal tractotomy/nucleotomy [11,12] that due to high risk of complications is reserved for patients with cancer-related pain and those with short life-expectancy, and (c) motor cortex stimulation (MCS) [13-15], which mechanism is not completely understood but which applicability for facial deafferentation pain has been repeatedly shown in multiple centers all over the world, including the United States.

Atypical facial pain (AFP), on the other hand, does not follow clear anatomical distribution, frequently crosses midline, and, not surprisingly, almost inevitably correlates with presence of psychological aberrations. Patients with AFP do not improve with surgical interventions; therefore, it is recommended to avoid surgery and continue treatment with anticonvulsants and antidepressants as soon as the diagnosis is made.

Other, less frequent pain syndromes, such as GPN [16], Eagle syndrome [17], Tolosa-Hunt, Raeder and Gradenigo syndromes, require completely different algorithm that also includes non-destructive and destructive options.

### Description of the algorithm (Figure 1)

**Figure 1:**
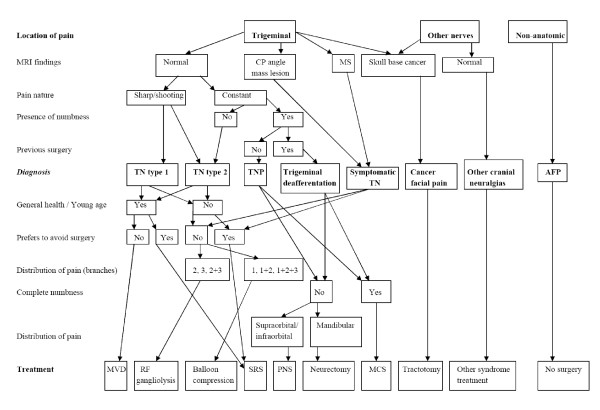
Flow diagram of the treatment algorithm.

1. If the patient has anatomical distribution of pain, determine if this distribution correlates with trigeminal territory or territory supplied by other nerves of face/head region (glossopharyngeal, n. intermedius, occipital); if it is limited to trigeminal area, proceed to step #2; if other nerves are involved, proceed with step #13; if pain is non-anatomical in character, proceed with step #14.

2. If the patient's pain is limited to trigeminal distribution, define if it has features of TN – sharp shooting (electric-shock like) pain, triggerable by touch, speech, cold wind, etc., responsive to oral carbamazepine, with spontaneous remissions, pain-free intervals – proceed with step #3; if any of the typical TN features are absent, but the majority of pain is sharp and shooting in nature, proceed with step #6; if the pain is in trigeminal distribution but is primarily constant and is associated with certain degree of sensory loss, proceed with step #9; obtain MRI of the brain in all of these cases to rule out mass lesions and demyelination.

3. If the patient has TN type 1 (typical TN) – as documented by TN features and brain imaging negative for mass lesions in the cerebellopontine angle and demyelination – the procedure of choice is *microvascular decompression *(MVD) through suboccipital craniotomy, independently of the trigeminal branch involved.

4. In the situation described in #3, if the patient cannot have MVD due to advanced age (older than 70) or severity of medical condition, **or **if the patient prefers to avoid open surgical intervention, **or **if the pain recurred after MVD performed in the past, **or **if the brain imaging is suggestive of demyelination (e.g., MS), **and **if the pain involves 3^rd^, 2^nd ^or 2^nd ^and 3^rd ^branches, the procedure of choice would be percutaneous stereotactic *radiofrequency gangliolysis*.

5. In all situations described in #4 **and **if the pain involves the 1^st ^branch distribution alone or in any combination with 2^nd ^and 3^rd ^branches, the procedure of choice is percutaneous trigeminal *balloon microcompression*.

6. If the patient has TN with atypical features, (some numbness, presence of constant pain in addition to sharp shooting sensation, pain is unresponsive to carbamazepine), the most likely diagnosis is TN type 2, and the algorithm from #3–5 applies, but the patient is informed about higher chance of complications, including facial numbness and pain recurrence.

7. If the patient's TN types 1 and 2 (typical or atypical TN) is associated with a mass lesion of the cerebellopontine angle, the surgery should address the mass lesion and the pain is expected to improve once the mass lesion is eliminated.

8. If the patient fits description of steps #3–6 **but **does not want to have any invasive intervention, the option of choice becomes *stereotactic radiosurgery *with Gamma Knife or, less often, linear accelerator; the patient is informed about destructive nature of radiosurgery and the lag between the treatment and onset of pain relief (1 to 6 months).

9. If the patient is diagnosed with the trigeminal neuropathic pain (**not **neuralgia), **and **maintains some degree of sensation in the area of pain, **and **the pain is localized to distribution of the supraorbital or infraorbital nerves, the treatment of choice is trigeminal branch stimulation with *peripheral nerve stimulation *technique.

10. If the patient fits description of step #9 **and **the pain is localized to distribution of any branches of the mandibular nerve, the treatment of choice is *neurectomy *or nerve decompression or non-surgical management.

11. If the patient has constant pain **and **complete numbness in the area of pain (anesthesia dolorosa) as a result of previous surgical deafferentation or stroke, **and **life expectancy more than 6 months, the treatment of choice is the contralateral *motor cortex stimulation *with epidural intracranial electrodes.

12. If the patient has trigeminal deafferentation pain with sensory impairment (anesthesia dolorosa), **or **any type of cancer-related pain in the area of face, **and **short (less than 6 months) life expectancy, the treatment of choice is percutaneous stereotactic *trigeminal tractotomy*.

13. If the patient's pain fits into category of other specific facial pain syndrome, such as glossopharyngeal neuralgia, cluster headaches, sudden unilateral neuralgiform pain with conjunctival injection and tearing (SUNCT), Eagle, Gradenigo, Tolosa-Hunt, Raeder, and other syndromes, a dedicated treatment modalities are considered outside of this algorithm.

14. If the patient is diagnosed with atypical facial pain, the *surgery is not recommended *and the patient is managed by combination of anticonvulsants and antidepressants.

## Results

Our algorithmic approach was used in 138 consecutive patients with medically intractable facial pain. The information about demographics, diagnoses and initial surgical interventions for these patients is summarized in Table 4. Clinical evaluations and diagnostic workups were followed in each case by the systematic choice of the appropriate intervention. The algorithm has proved easy to follow, and the recommendations include the identification of the optimal surgery for each patient with other options reserved for failures or recurrences. For example, among these patients almost two thirds (62%) had trigeminal neuralgia, majority of which may be classified as a TN type 1 (typical idiopathic TN), and the remaining group was divided between the symptomatic TN (due to neoplasms and multiple sclerosis) and TN type 2 (atypical idiopathic TN). Only few patients had other cranial neuralgias and syndromes, and the incidence of TNP and AFP was about 10% each.

**Table 4:** Demographic characteristics of 138 consecutive patients presenting with medically-intractable facial pain

**Age (years)**	20–94 (mean – 58)
**Gender**	95 women, 43 men
**Side of pain**	69 right, 63 left, 6 bilateral
**Diagnosis**	
Trigeminal neuralgia type 1	47
Trigeminal neuralgia type 2 (Atypical TN)	24
Post-traumatic trigeminal neuropathic pain	11
Post-surgical deafferentation trigeminal pain	11
Post-stroke trigeminal pain	4
Symptomatic TN (multiple sclerosis)	8
Symptomatic TN (cerebellopontine lesions)	6
Other cranial nerve involvement	10
Cancer-related facial pain	2
Atypical facial pain	14
**Initial procedure (after failure of medical management)**	
MVD	32
RF gangliolysis	28
Balloon compression	5
SRS	7
PNS	11
Neurectomy	4
MCS	3
Interventions on other nerves	9
No surgery	39

The number of MVD and RF gangliolyses was approximately the same; RF gangliolysis was done as a repeat procedure for pain recurrence in 9 cases after previous RF ganglilolysis and in 3 cases after previous MVD. Balloon compression was used only on five occasions and only in patients with TN and pain involving 1^st ^branch distribution. Relatively recent acquisition of Gamma Knife is reflected by its relative underutilization in our practice, but this modality is expected to be used in about 25% of all TN patients.

PNS approach [9,10] was used only in patients with TNP due to post-traumatic or post-surgical neuropathy (trigeminal deafferentation pain). MCS [15] was used in those with facial pain after thalamic infarctions and with anesthesia dolorosa (an extreme case of trigeminal deafferentation pain developed as a complication of previous interventions for treatment of TN). Computed tomography (CT)-guided trigeminal tractotomy is considered only in patients with short life expectancy and is rarely performed.

Patients with atypical facial pain (AFP) were not offered any surgical intervention, mainly on the basis of its ineffectiveness in suppressing AFP symptoms and risk of unwarranted complications. The patients understood the rationale for avoiding the surgery and proceeded with medical treatment, usually including anticonvulsant and antidepressant medications. In addition to this, several other patients with different pain conditions were advised against surgery or chose not to have operations for various reasons.

From all patients followed for their facial pain, with the exception of those with AFP and those that underwent Gamma Knife SRS, 95.8% obtained pain relief upon discharge from the hospital

## Conclusion

The treatment algorithm for the intractable facial pain described in this paper appears to be effective for patients with a wide variety of painful conditions involving the face. It applies to vast majority of patients presenting to a neurosurgical clinic and is associated with extremely high degree of pain relief and patient satisfaction. Therefore, in our opinion, it may be recommended for use in other institutions for subsequent validation and wider adoption.

## Abbreviations

AFP – atypical facial pain; ATN – atypical trigeminal neuralgia; CT – computed tomography; GPN – glossopharyngeal neuralgia; MCS – motor cortex stimulation; MRI – magnetic resonance imaging; MS – multiple sclerosis; MVD – microvascular decompression; PNS – peripheral nerve stimulation; RF – radiofrequency; SRS – stereotactic radiosurgery; SUNCT – sudden unilateral neuralgiform pain with conjunctival injection and tearing; TMJ – temporomandibular joint; TN – trigeminal neuralgia; TNP – trigeminal neuropathic pain.

## Authors' contributions

KVS conceived the article, treated all patients and wrote the article; HN, MEC and NM collected the data, participated in data analysis, and participated in manuscript drafting. All authors read and approved the final manuscript.
